# Assessment of Determinants of Dietary Vitamin D Intake in a Polish National Sample of Male Adolescents

**DOI:** 10.3390/nu17122024

**Published:** 2025-06-17

**Authors:** Małgorzata Stachoń, Katarzyna Lachowicz

**Affiliations:** Department of Dietetics, Institute of Human Nutrition Sciences, Warsaw University of Life Sciences (SGGW-WULS), 159c Nowoursynowska Street, 02-776 Warsaw, Poland; katarzyna_lachowicz@sggw.edu.pl

**Keywords:** calciferol, dietary sources, fish, male post-primary students, Poland

## Abstract

Background/Objectives: Calcitriol, the active form of vitamin D, has a broad physiological effect, and its deficiency has been identified as a risk factor for many diseases. This study aimed to analyze the dietary intake of vitamin D and the factors determining its intake among Polish post-primary school students. The data obtained were then related to the dietary recommendations for the Polish population. Methods: The study was conducted on a nationwide sample of 3257 male adolescents (aged 14–20 years) recruited from all macroregions of Poland. Dietary vitamin D intake (dVDi) was assessed using the Vitamin D Estimation Only–Food Frequency Questionnaire (VIDEO-FFQ). Results: The median dietary intake of vitamin D was 4.36 µg daily. This value was below the recommended intake of 15 µg of vitamin D, according to Polish standards, in almost 80% of the study group. The observed dietary vitamin D intake bellow the recommended level ranged from 35.5% of students attending schools in the North-Western macroregion to 93.7% in the Central macroregion, from 45.4% of students attending schools located in the countryside to 92.7% in big cities, from 85.3% among underweight students to 76.7% of obese students, over 77% in both age groups (14–17 and 18–20 years old), and over 78% in both groups: supplementing and not supplementing vitamin D. Fish and fish products provided the highest vitamin D (38.7%), while cereal products and fats provided the lowest (4.49% and 4.35%, respectively). The highest amounts of vitamin D were provided by salmon, rainbow trout, herring, and eel (fish species containing 7–15 µg of vitamin D in 100 g of product), and halibut, mackerel, brook trout, sole, and tuna (fish species containing 1.05–4 µg of vitamin D in 100 g of product), and these fish were consumed in the largest quantities by male adolescents. Dietary vitamin D intake was notably higher in adolescents from the North-Western macroregion of Poland (median: 50.57 vs. 3.72–5.18 µg daily for other macroregions), those attending schools in the countryside (median: 49.49 vs. 3.97–4.39 µg daily for other locations of the school), those with a normal body weight (median: 4.59 vs. 3.38 µg daily for adolescents with underweight), and those who took vitamin D supplements (median: 4.71 vs. 4.06 µg daily for adolescents not supplemented with vitamin D). However, the results showed that dVDi was not dependent on age. Conclusions: The study results indicate that low dVDi among Polish male adolescents can be attributed to the limited supply of vitamin D from dietary sources, especially fish and fish products. The necessity for interventions has been identified, including nutritional education on the role of vitamin D and its sources in the diet.

## 1. Introduction

Chemically, calcitriol is 9,10-seco(5Z,7E)-5,7,10(19)-cholesta-triene-1α, 3β, 25-triol (to see the structural formula, check [1]). The other name frequently used for calcitriol is 1,25-dihydroxyvitamin D (1,25(OH)_2_D_3_). It is the biologically active form of vitamin D (D3—cholecalciferol and D2—ergocalciferol) and has been demonstrated to exhibit a number of physiological functions (pleiotropic effect) at both the genomic level (through activation of the vitamin D receptor, VDR, and regulation of gene expression) and the extra-genomic level. The importance of vitamin D for calcium-phosphate homeostasis and bone health is well-documented. However, its influence on other systems, including the immune, nervous, muscular, cardiovascular, gastrointestinal, excretory, reproductive and respiratory systems, is equally significant. A deficiency in vitamin D has been demonstrated to increase the risk of a number of non-communicable diseases, including autoimmune diseases, osteoporosis, type 2 diabetes, anemia, depression and certain types of cancer [2,3,4,5,6].

Vitamin D deficiency (VDD), characterized by a total blood concentration of 25-hydroxyvitamin D (25(OH)D) ≤ 50 nmol/L (≤20 ng/mL), has become a prevalent condition, particularly among individuals residing at higher latitudes (above the 37th parallel), where the cutaneous synthesis of this vitamin is inadequate or practically non-existent (in Poland, this occurs from October to March) [7,8,9,10,11].

A group that is particularly at risk of developing hypovitaminosis D and its ensuing health consequences, including in later life, is school-aged adolescents [9,12,13,14,15,16]. The global prevalence of VDD among adolescents ranges from 4% to 91%, which is considered a serious public health problem even in regions with high sun exposure [17]. A study conducted among children and adolescents aged 3–18 years from the Central Poland region showed a high prevalence of VDD, ranging from 10.7% to 80.4% (in August and in January, respectively), accompanied by a low percentage of normal serum 25(OH)D concentrations (from 3.6% in January to 42.1% in August). The study revealed that the median 25(OH)D concentrations remained below the lower limit of the optimal range throughout the year [18].

The reasons for the increased risk of VDD among Polish adolescents are multiple, including a reduced time spent outdoors from April to the end of September, low exposure to sunlight, low dietary vitamin D intake (dVDi), and a lack of recommended vitamin D supplementation [11,19]. Factors that may contribute to reduced sun exposure and outdoor activity in this age group (even during the more than two-month holiday period, which should be conducive to outdoor activities) include an increase in screen time, high academic pressure, parental concerns about the safety of children and adolescents, a shift to more structured extracurricular and leisure activities, and photoprotective practices [20,21,22].

Taking into account the above considerations and the fact that increasing one’s daily dVDi intake may contribute to a reduction in VDD, especially during autumn and winter months, an attempt was made to assess vitamin D intake from dietary sources and its determinants in a cohort of male adolescents attending Polish post-primary schools. The data obtained were then related to the vitamin D intake recommendations in Poland.

This study contributes to the extant body of knowledge concerning the intake of vitamin D in male adolescents, a demographic that has been underrepresented in the majority of studies to date, which have predominantly focused on young women and female adolescents.

## 2. Materials and Methods

This study was carried out at the Department of Dietetics, Warsaw University of Life Sciences (WULS-SGGW), and was conducted according to the guidelines established by the Declaration of Helsinki. The Ethics Committee of the Central Clinical Hospital of the Ministry of Interior and Administration in Warsaw (2/2021) approved all procedures involving human participants.

The data for the study were collected from 1 February 2022 to 31 March 2022.

### 2.1. Study Design

A cross-sectional study was conducted on a nationwide sample of post-primary school students attending the following types of schools: first-degree vocational schools, general secondary schools, and technical schools.

The list of schools for the sample was obtained from the online National Register of Schools and Educational Institutions of the Ministry of National Education [23].

The selection of schools for the study took into account the fact that the territory of Poland is divided into seven macroregions that group voivodeships into sixteen basic administrative units (voivodeships) (NUTS 1 units in the statistical division of Poland from the year 2021 [24]). This division was made taking into account historical, cultural, economic and geographical factors. Additionally, each voivodeship was divided into counties.

The selection of study participants was based on a stratified random selection of schools, with the objective of obtaining a representative sample of all Polish macroregions: the Masovian Voivodeship, the Central region (in which the Łódź and Świętokrzyskie voivodeships are located), the Eastern region (in which the Podkarpackie, Lublin and Podlaskie voivodeships are located), the Northern region (in which the Kuyavian–Pomeranian, Warmia–Masurian and Pomeranian voivodeships are located), the North-Western region (in which the Greater Poland, Lubusz and West Pomeranian voivodeships are located), the Southern region (in which the Lesser Poland and Silesian voivodeships are located) and the South-Western region (in which the Lower Silesian and Opole voivodeships are located). The present study employs a sample selection procedure consistent with that of other studies carried out in Poland [25,26,27].

The sample selection was executed in the following stages: (1) schools were selected from counties, and (2) counties were selected from provinces. In the first step, a stratified sampling of schools was conducted for all macroregions, i.e., 10 counties were randomly selected from each province of the country (which resulted in 160 counties). In the second step, 10 post-primary schools were randomly selected from all counties (which resulted in 1600 post-primary schools). Additionally, schools from neighboring counties were selected if there were not enough schools in the selected county. A total of 1600 post-primary schools were invited to participate voluntarily in the study by contacting the principals of these schools. The objectives of the study and its protocol were communicated to the principals, who were subsequently provided with an electronic link to the questionnaire. Principals who consented to the participation of their school’s pupils in the survey were asked to provide pupils with an electronic version of the questionnaire. Furthermore, participation in the survey was entirely voluntary, with no coercion exerted on the pupils. Written informed consent was obtained from all parents or legal guardians of the participants, authorizing their children’s involvement in the study. The survey form was completed anonymously, thus preventing the collection of data that would allow for the identification of the respondent.

The students were informed about the purpose and assumptions of the study by the principal of the school they were attending. The students completed the survey during lessons at school under the supervision and support of teachers appointed by the school principal. These appointed teachers conducted the lessons during which the students completed the survey, supervised the course of completing the survey and additionally ensured that the answers entered by the students were consistent with the actual state of affairs.

Students from 67 schools responded to the invitation to participate in the study. The survey was completed and returned by 7947 post-primary school students.

### 2.2. Study Participants

The following inclusion criteria were adopted for the study:

− being a student of a school whose principal agreed to participate in the study,
− being aged 14–20 (typical age for this level of education in Poland),
− participants and parents or legal guardians providing informed consent to participate in the study.

The following exclusion criteria were adopted for the study:

− female gender,
− incompletely completed survey and unreliable data included in the survey (e.g., regarding body weight and/or height, number of servings of consumed products and/or product groups).

The objective of the present study was to reach and examine the general population of Polish male post-primary school adolescents and to assess vitamin D intake from dietary sources and analyze the determinants of this intake, regardless of health status. Consequently, the exclusion criteria did not encompass, inter alia, co-occurring diseases or medications that could potentially affect the level of consumption of fish and other dietary sources of vitamin D.

Taking into account the above inclusion and exclusion criteria from the study, the research group consisted of 3257 students from 64 upper post-primary schools from all provinces in Poland.

**Figure 1:**
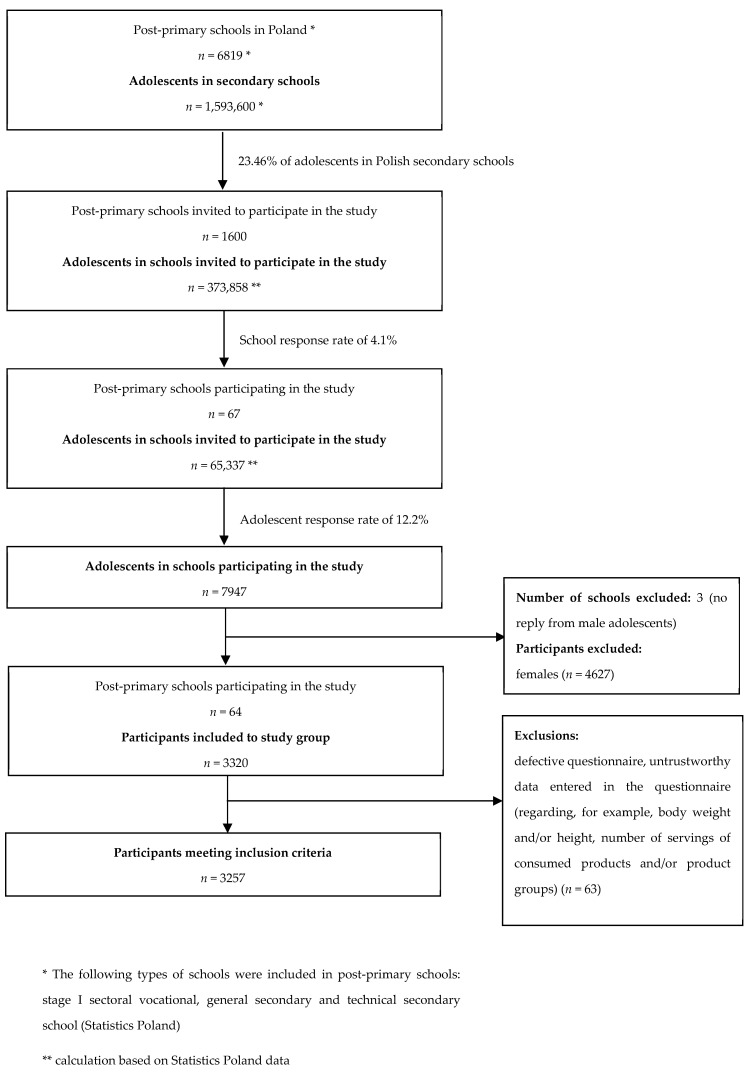
The sampling and recruitment procedure for the study group.

The procedure for selecting participants and recruiting them for the study group is presented in Figure 1.

### 2.3. Questionnaire

The data presented in this study was collected using the computer-assisted online interview method (CAWI) [28].

The questionnaire employed in this study incorporated questions that served to verify the criteria for switching on and exclusion, i.e., sex, age and school name in a given province and the county.

Additionally, the questionnaire incorporated inquiries pertaining to the utilization of vitamin D supplements, which were appraised by study participants on a self-declaration basis (i.e., either ‘yes’ or ‘no’).

The applied VIDEO-FFQ (Vitamin D Estimation Only-Food Frequency Questionnaire), which was an indispensable component of the questionnaire employed in the present study, has been developed for use in the Polish population to assess vitamin D intake from dietary sources. This VIDEO-FFQ was developed and has previously undergone validation in a group of young Polish women and, after adjusting for the Croatian population, also in a population of Croatian women [29,30]. Afterwards, it was used in multiple studies including populations of female individuals [26,31] and male individuals [32,33].

VIDEO-FFQ is a rapid and efficient instrument that exhibits high validity and repeatability. The evaluation of vitamin D intake from food in large populations is facilitated by this method. The instrument has been included in the Registry of Validated Brief Dietary Assessment Instruments by the National Institutes of Health (NIH)-National Cancer Institute of the United States of America [34].

The VIDEO-FFQ questionnaire utilized in this study incorporated inquiries pertaining to the consumption of specific food products in the year prior to the survey, irrespective of the season. Respondents were requested to specify the number of servings per month or week, contingent on the product, in response to open-labeled questions. The original VIDEO-FFQ version has been placed in the Supplementary Materials (Figure S1: VIDEO-FFQ-Vitamin D Estimation Only-Food Frequency Questionnaire).

The participants were invited to provide the number of products consumed and added to meals, while they could indicate the number of servings not only as whole numbers but also as decimal parts of a serving [29]. The mean daily intake of vitamin D from food was calculated for each participant based on the collected data, using formulas developed for the VIDEO-FFQ. The VIDEO-FFQ questionnaire was based on the assessment of the frequency of food consumption, taking into account only food products that are a source of vitamin D in the amount of at least 0.01 μg/100 g, which were divided into eight groups. The construction of the questionnaire was based on (1) Polish Tables of Food Composition, which were developed based on the reference chemical analysis of food products available on the Polish market [35], which allowed us to determine the content of vitamin D, as well as (2) the Polish atlas of portion sizes of food products and dishes [36], which allowed us to determine the size of the portion.

The method used to analyze the answers entered by the study participants in the questionnaire is as follows: (1) the total number of servings was divided per seven or per 30 days, in the case of products specified weekly or monthly; (2) the vitamin D intake from each product was estimated using the following equation: vitamin D intake (μg) = daily number of servings × typical vitamin D content in 1 serving; (3) the total daily dietary vitamin D intake was obtained as the sum of the vitamin D intake values from all the analyzed groups of products.

The procedure used allowed the calculation of the total vitamin D intake from the diet, as well as the vitamin D intake from specific groups of products (GR), including (1) fish and fish products (F/FP: F/FP_GR1: salmon, rainbow trout, herring, eel; F/FP_GR2: halibut, mackerel, brook trout, sole, tuna; F/FP_GR3: cod, flounder, plaice, pollock, hake; F/FP_GR4: herring, sardine, and tuna products; F/FP_GR5: other fish products); (2) dairy products; (3) eggs; (4) meat and meat products; (5) cereals; and (6) fats [35].

A 15 µg cut-off point was utilized in order to compare the obtained data with the Polish guidelines for the adequate intake of (AI) vitamin D per day [37].

The body mass index (BMI) was computed on the basis of the participants’ self-reported body weight in kilograms and height in meters, with the Quetelet equation (body weight/height^2^) being utilized for this purpose. The OLAF program (“Development of the reference range of blood pressure for the population of children and adolescents in Poland—PL0080 OLAF”) [38], based on Polish reference growth curves, taking into account gender and age [39], was used to assess BMI in the age group of 14–18 years, while the World Health Organization classification [40] was used to assess BMI in adults (19–20 years).

### 2.4. Statistical Analysis

The Shapiro–Wilk test was employed to verify the normality of the distribution of the obtained data.

In light of the non-parametric distributions, a range of statistical analyses, including the U Mann–Whitney and the Kruskal–Wallis tests, were employed to compare subgroups and the Spearman rank coefficient was used to analyze correlations. Statistica package, version 13.3 (TIBCO Software Inc., San Ramon, CA, USA), was used for the statistical analysis. The significance level was set at *p* ≤ 0.05.

For the subsequent subgroup analyses, the entire sample was divided into the following subgroups, based on:

(a) Age: from 14 to 17 years old and from 18 to 20 years old;
(b) BMI: underweight, normal weight, overweight and obesity—for adults, the standard cut-offs established by WHO were applied as <18.5 kg/m^2^ for underweight, 18.5–24.9 kg/m^2^ for normal weight, 25–29.9 kg/m^2^ for overweight, and ≥30 kg/m^2^ for obesity [38], while for minors the Polish growth reference cut-offs were applied [38] as <5th percentile for underweight, 5th–85th percentile for normal weight, 85th–95th percentile for overweight, and ≥95th percentile for obesity [30];
(c) Location of the school attended by the study participants: countryside, small city (less than 20,000 inhabitants), medium city (from 20,000 to 100,000 inhabitants) and big city (over 100,000 inhabitants);
(d) Macroregion in which the school attended by the study participants was located, defined on the basis of the categories of macroregions adopted by the Central Statistical Office in Poland: North-Western, Northern, Eastern, South-Western, Southern, Central, and Masovian [24];
(e) Declaration of taking vitamin D supplements: no or yes;
(f) Vitamin D sources: fish and fish products depending on their content (F/FP_GR1: 7–15 µg per 100 g of product, F/FP_GR2: 1.05–4 µg per 100 g of product, F/FP_GR3: 0.35–0.5 µg per 100 g of product, F/FP_GR4, F/FP_GR5), and other food groups: dairy products, eggs, meat and meat products, cereals, and fats.

## 3. Results

### 3.1. Characteristics of the Research Group

The general characteristics of the studied group of participants (by macroregion of Poland, school location, age, BMI classification, and vitamin D supplementation) are shown in Table 1.

The highest percentage of male adolescents hailed from the North-Western macroregion (approximately 33%), followed by the Eastern (approximately 18%) and Southern (almost 17%) macroregions. In comparison, the lowest percentage hailed from the Central and Mazovian voivodeship macroregions (6% each). The most significant number of respondents attended schools in medium-sized cities (66%), while the smallest number attended schools in rural areas (less than 4%). Most participants were between 14 and 17 years old (3/4 of the participants), with the remaining 1/4 being of legal age (18–20 years). Approximately 70% of participants in both age groups were of normal weight, while a larger proportion of participants were not taking vitamin D supplements (54%).

### 3.2. Dietary Intake and Sources of Vitamin D

**Figure 2:**
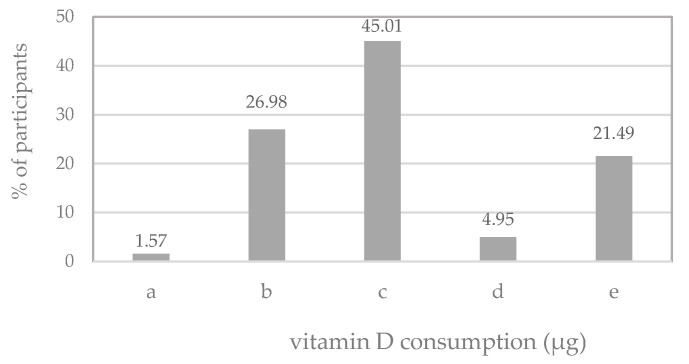
Percentage of male adolescents with vitamin D dietary intake: 0 µg per day (a), (0, 2.5) µg per day (b), 〈2.5, 10) µg per day (c), 〈10, 15) µg per day (d) and ≥15 µg per day (e).

The median dVDi in the studied group of Polish male adolescents reached 4.36 µg daily, with a range of 0.00 µg to 72.59 µg daily (Table 2). This intake was lower than the recommended daily intake of 15 µg in Poland [37], which was met by only 21% of male students. Furthermore, it was observed that approximately 74% of the participants had a dVDi below 10 µg (lower than the EAR, i.e., Estimated Average Requirement—in many European countries [41]), and 29% had a dVDi below 2.5 µg (below the lower LRNI, i.e., Limit of Reference Intake value [41]) (Figure 2).

The observed deficiency of vitamin D intake below the recommended level (15 µg per day) ranged from 35.48% of students attending schools in the North-Western macroregion to 93.69% in the Central macroregion, from 45.38% of students attending schools located in the countryside to 92.73% in big cities, and from 85.28% of underweight students to 76.7% of obese students. In both age groups (14–17 years and 18–20 years), the prevalence of this deficiency was similar (78.24% and 77.12%, respectively). Similarly, in the groups of male adolescents who took vitamin D supplements and those who did not take this supplement (78.64% and 78.57%, respectively), the prevalence was also comparable (for details see Figure S2 in Supplementary Materials).

The data pertaining to the intake of vitamin D from diverse food groups (comprising various species of fish and fish products) among the male post-primary school students in this study is delineated in Table 2.

The median dVDi was documented to be highest for fish and fish products (1.13 µg daily), followed by meat and meat products (0.71 µg daily), eggs (0.61 µg daily), and dairy products (0.39 µg per day), while it was lowest for cereals (0.13 µg daily) and fats (0.05 µg daily). Dietary vitamin D intake ranged from 0.00 μg to 67.53 μg for fish and fish products, 20.15 μg for dairy products, 8.71 μg for eggs, 6.15 μg for meat and meat products, 2.28 μg for cereals, and 3.07 μg for fats. The highest median dVDi from various species of fish and fish products was reported for F/FP_GR2, which included halibut, mackerel, brook trout, sole and tuna.

**Figure 3:**
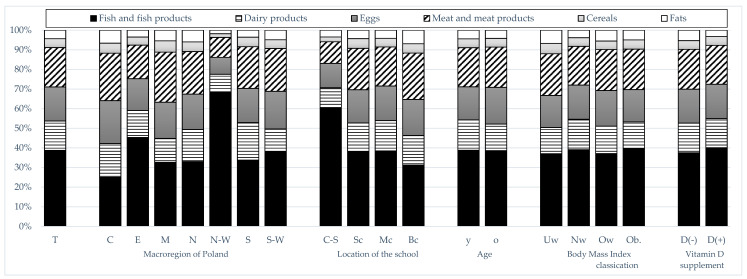
Dietary vitamin D sources in the subgroups of male adolescents. (T—total, C—Central, E—Eastern, M—Masovian Voivodeship, N—Northern, N-W—North-Western, S—Southern, S-W—South-Western; C-S—countryside, Sc—small city, Mc—medium city, Bc—big city; y—14 to 17 years old, o—18 to 20 years old; Uw—underweight, Nw—normal weight, Ow—overweight, Ob.—obesity; D(−)—not supplementing vitamin D, D(+)—supplementing vitamin D).

In contrast, the percentage contribution of different food groups to the daily vitamin D supply was as follows: fish and fish products constituted 38.7% of the vitamin, meat and meat products constituted 20.08%, eggs constituted 17.37%, dairy products constituted 15%, cereals constituted 4.49%, and fats constituted 4.35%. A significantly higher percentage of fish in the supply of vitamin D was noted for participants from the North-Western macroregion, followed by the Eastern macroregion, as well as for students from schools located in rural areas (Figure 3).

The quantity of servings of F/FP and other products consumed per week or per day, as declared by participants, is shown in Table 3.

The median intake of F/FP amounted to 2.10 servings per week, ranging from 0.93 to 50.63 per week. The highest number of servings was reported for F/FP_GR1, including salmon, rainbow trout, herring, and eel (0.23 servings per week). For the other food groups, the consumption of cereals and dairy products was higher (about three servings per day each).

**Figure 4:**
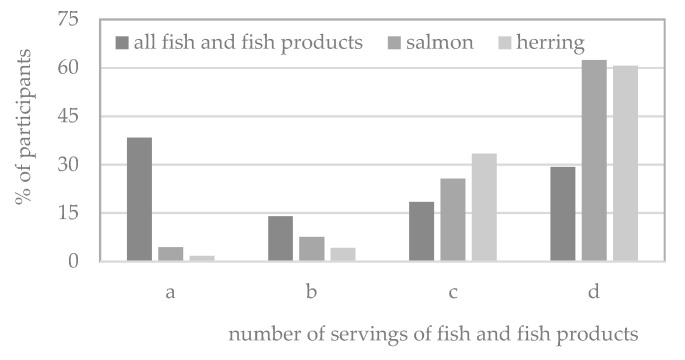
Percentage of male adolescents consuming: ≥2 (a), 〈1, 2) (b), and (0, 1) (c) servings or not consuming (d) fish (including salmon and herring) and fish products.

As demonstrated in Figure 4, the data indicate that 29.9% of the study’s participants declared zero fish and fish product consumption. Conversely, 38.4% of the participants consumed at least two servings of fish weekly. The remaining 18.4% consumed no more than one serving, and 13.9% consumed between one and two servings.

As illustrated in Figure 4, salmon and herring were consumed by 38% and 39% of the male participants surveyed, respectively. However, these fish were not the source of the highest amount of vitamin D in food (as demonstrated in Table 2, the median intake was 0.00 µg for both fish species). The median number of servings consumed per week was 0.00 for both considered fish species (see Table 3).

### 3.3. Determinants of Dietary Intake of Vitamin D

#### 3.3.1. Macroregions

Table 4 and Table S1 (in Supplementary Materials) compare dVDi, including its consumption from different sources, in the subgroups of adolescents from different macroregions.

The study demonstrated that participants from North-Western Poland exhibited the highest total dVDi and total vitamin D supply from fish and fish products. This was determined by the highest supply of F/FP_GR2 fish. Moreover, male students from Eastern Poland had a higher dVDi (from all food groups, including F/FP (*p* < 0.0001) and F/FP_GR2 fish (*p* < 0.0001)) compared to their counterparts from other macroregions. However, the overall dietary intake of vitamin D exhibited no statistically significant disparities among the other macroregional subgroups. Regional variation was observed in the contribution of other food groups to vitamin D provision (dairy products, *p* < 0.0001; eggs, *p* < 0.0007; meat and meat products, *p* < 0.0001; cereals, *p* = 0.011 and fats, *p* = 0.0002) (see Table S1 in Supplementary Materials and Figure 3 for details).

#### 3.3.2. Location of the School

As illustrated in Table 4 and Table S2 (in the Supplementary Materials), a comparison of dVDi, including its intake from different sources, was performed in male adolescents divided according to their school location.

The present study found that pupils from rural schools consumed higher levels of vitamin D than their urban counterparts. Furthermore, pupils from medium-sized cities demonstrated higher levels of dVDi compared to their big city school peers.

The location of the school determined the intake of F/FP (*p* = 0.0014), F/FP_GR2 (*p* < 0.0001) and F/FP_GR5 (*p* = 0.002) (for details, see Table S2 in the Supplementary Materials).

#### 3.3.3. Age

A comparison of dVDi, including its intake from different sources, across age subgroups is shown in Table 4 and Table S3 (in the Supplementary Materials).

Age did not affect the total dVDi or its basic sources in food (fish and their products, meat, dairy, cereals, and fats). Contrary, older participants consumed more vitamin D from eggs (*p* < 0.0001).

#### 3.3.4. Body Mass Index Classification

The following section presents a comparison of dVDi, including its consumption from different sources, in the subgroups of underweight, normal weight, and a condition of excessive body weight (i.e., overweight and obese) male adolescent participants (see Table 4 and Table S4 (in the Supplementary Materials)).

The analysis revealed that the total intake of vitamin D, as well as its intake from eggs, was higher in students with a normal weight compared to students who were underweight (*p* = 0.022). In the present study, it was found that dVDi from dairy products in normal-weight study participants was higher than in the other subgroups (*p* < 0.0001). In the case of subjects who were categorized as being overweight and obese, the intake of vitamin D from fat sources was found to be lower than other subjects (*p* = 0.0002). An analysis of the data revealed no statistically significant differences in dVDi among the study groups, which included participants who consumed F/FP, meat and meat products, and cereals.

#### 3.3.5. Declaration of Taking Vitamin D Supplements

Table 4 and Table S5 (in the Supplementary Materials) compare dVDi from different sources in the subgroups of adolescent boys who did not take supplements and those who did.

The investigation revealed that participants who did not supplement their diet with vitamin D had lower dVDi than those who supplemented. Furthermore, individuals who received vitamin D supplements exhibited higher dVDi from F/FP (*p* = 0.0002), dairy products (*p* < 0.0001), eggs (*p* = 0.0002), meat and meat products (*p* = 0.0003), and cereals (*p* = 0.001). After a comprehensive analysis, it was determined that there were no discrepancies in the dVDi obtained from fats between the subgroups that did and did not receive vitamin D supplements. Furthermore, the supplementation subgroup demonstrated higher levels of dVDi from one to three groups of fish (F/FP_GR1, *p* < 0.0002; F/FP_GR2, *p* < 0.0001; F/FP_GR3, *p* = 0.01). The present study found no significant differences in dVDi from F/FP_GR4 and F/FP_GR5 between the non-supplementing and vitamin D supplementation subgroups.

### 3.4. Correlations

Table 5 presents the results of the analysis of the correlation between the declared number of weekly servings of fish and fish products and the dVDi from them, and the BMI and total dVDi in the group of male adolescents.

There was a statistically significant positive correlation between total dVDi and the intake of fish and fish products and the intake of vitamin D from these sources. The strongest relationships were identified for F/FP_GR1 and F/FP_GR2 fish and fish products, respectively.

No significant correlation was observed between total dVDi and BMI.

## 4. Discussion

The current study appraised dietary vitamin D intake from differing sources in a population of Polish male adolescents, finding that the median dVDi was low (4.36 µg per day) and significantly lower than the Polish recommendations (15 µg per day). Almost 80% of respondents had a supply of vitamin D that was too low. The implementation of the recommended dVDi was 29.3%. Furthermore, the study demonstrated that dVDi was notably higher in adolescents from the North-Western macroregion of Poland, those attending schools in rural areas, those with a normal body weight, and those who took vitamin D supplements. Vitamin D intake was positively correlated with the consumption of fish and fish products, but did not correlate with BMI.

Vitamin D intake in the examined group of male adolescents was almost twice as high as in female adolescents (median 2.33 µg per day) in a similar population-based cohort study among Polish post-primary school students aged 14–20 years, in which the same FFQ questionnaire was used to assess vitamin D intake [26]. In the same study, a higher percentage of girls (98%) had insufficient vitamin D intake, and the attainment of sufficient vitamin D intake according to Polish standards was at a lower level (15.5%).

The higher vitamin D supply in our study group of male adolescents was due to a higher intake of fish (1.13 µg daily), meat and meat products (0.7 µg daily), and eggs (0.61 µg daily) compared to female adolescents (0.52, 0.39, and 0.38 µg daily) [26].

In other cross-sectional study carried out in Poland on groups of 13–15-year-old boys from sport schools, dietary vitamin D intake was lower (mean ± SD: 3.2 ± 1.5 µg, range: 0.9–8.3 µg) than in our study. A deficient intake of vitamin D was found in all students [42]. A lower vitamin D supply (2.8 ± 0.8 µg) was also observed in the Warsaw Population Screening Study, which involved Polish boys aged 16.05 ± 1.0 years [43].

The results of other studies conducted among Slovenian adolescents aged 10–19 years and included in the systematic review also showed low vitamin D intakes among male teenagers (averaged between 1.4 and 4 µg per day) [44]. In a nationally representative Slovenian study, which did not take into account the amount of supplementation, the median daily vitamin D intake among male adolescents (aged 10–17 years) was lower (2.7 µg) [45] than in our study. A lower intake vitamin D was also found in another earlier study that included teenage populations (14–18 years old) from Central-Eastern European countries, including Poland. The lowest intake was recorded among young men from Austria (1.74 ± 0.98 µg), followed by Poland (2.92 ± 1.31 µg) and Hungary (3.13 ± 1.4 µg); intake was higher in Slovenia (3.72 ± 3.26 µg) [46].

A Slovenian study of male adolescents aged 15–16 years [47] and the D-VinCHI (D Vitamin in Children) study, which investigated the vitamin D status of school children aged 4–11 years in Northern Ireland [48], found vitamin D intakes from food (4.0 and 4.4 µg per day, respectively) that were comparable to those of Polish adolescents.

A vitamin D intake higher than that in the present study was observed for Sweden male adolescents aged 11–18 years old (5.8–7.6 µg and 8.2 µg) [49,50] and Finish adolescents aged 15–17 years attending 8-year examinations for the PANIC (Physical Activity and Nutrition in Children) Study (11.7 µg per day) [51]. This differentiation is confirmed by a review of European studies on vitamin D intake that showed higher intakes in Northern Europe (up to 11 µg per day) than Central and Southern Europe [14,41]. This can be explained by the high consumption of sea fish and enriched foods (especially dairy products) in some Nordic countries [49,50,51]. In Poland, the only products for which vitamin D enrichment is mandatory are bread spreads (with the exception of butter) [52].

The respondents whose schools were located in the North-Western and Eastern macroregion, as well as in rural areas, and those declaring the consumption of dietary supplements and foods containing vitamin D consumed a greater total amount of this vitamin compared to the other respondents.

The higher dVDi in the North-Western macroregion is likely to have been influenced by two key factors. The first is the macroregion’s proximity to the Baltic Sea and second is its abundance of lake districts, which results in a high availability of various species of F/FP. A similar situation was observed in the Eastern macroregion, which is associated with the presence of lake districts. As was evidenced in our previous research, the total vitamin D intake of female adolescents from schools in the Northern region exceeded that of their counterparts from Eastern schools [26].

A somewhat unexpected finding was that male adolescents attending schools in the countryside exhibited higher levels of dVDi compared to their counterparts from schools in small, medium, and big cities.

As indicated by the testimonies of male adolescents from schools located in the North-Western and Eastern macroregions, as well as those from rural areas, the primary sources of vitamin D were found to be fish and fish products, predominantly from F/FP_GR2: halibut, mackerel, brook trout, sole, and tuna. In the majority of cases, these are not species of fish that are characteristic of Polish lakes and the Baltic Sea. However, it is possible that the element of tradition and/or habit pertaining to the consumption of larger fish was significant in this instance.

In a separate study [27] that was conducted among secondary school students in Poland, no differences in the consumption of fish and fish products resulting from place of residence (both region and size) were found. This is likely to be the reason for the lack of differences in vitamin D intake from food products.

The utilization of dietary supplements containing vitamin D by the studied adolescents may signify a heightened awareness of the potential benefits of such supplements, both for themselves and for their parents and legal guardians. This could have had an impact on the higher intake of vitamin D from food products in general, as well as from the primary source, i.e., fish.

In a study involving children aged 4–11, it was demonstrated that children who consumed supplements and obtained vitamin D from food products exhibited sufficient levels of vitamin D in their blood compared to children who had insufficient levels of vitamin D in their blood. Consequently, the ingestion of vitamin D supplements is likely to be associated with an increased consumption of dietary sources of this vitamin [48].

However, it is noteworthy that, in our own study, just over 50% of the study participants (54.01%) declared taking supplements, which suggests that a significant proportion of the study participants are unaware of or ignore the benefits of using supplements. A comparable prevalence of vitamin D supplementation was identified in a previous study: only half of the female adolescents surveyed reported taking supplements [26]. In a study by Glatt et al. (2022) [48], 42% of children did not take supplements. The authors reported that in this case, parents may not have been aware of the insufficient supply of this vitamin because they believed that their children were getting enough vitamin D. The authors claimed that the parents were not aware of the benefits of supplementation, did not know what supplement they could use, forgot to supplement, or were simply not determined to do so. Another Finnish study found that about 29% of all adolescents surveyed (about 28% of male adolescents) declared that they did not take vitamin D supplements [51].

In this study, male adolescents who were underweight, as determined by their BMI, exhibited a lower intake of vitamin D compared to their peers with a normal weight. It is noteworthy that this discrepancy was not attributable to a reduced dVDi from fish and fish products; rather, it was attributed to a lower intake of vitamin D from dairy products and eggs. In the preceding study, no disparities in total vitamin D intake or its intake from all food product groups were identified between BMI subgroups [26].

Since the ability to synthesize sufficient amounts of vitamin D dermally is limited by various factors, in order to maintain normal serum vitamin D concentrations in adolescents in Poland, it may be important to increase the intake of foods that are a significant source of this vitamin, especially in autumn and winter. However, it should be borne in mind that the range of foods naturally containing vitamin D is limited (fish and eggs, and to a lesser extent milk and dairy products), and that this is not able to ensure the same vitamin D concentrations in the blood as in the summer months. This must therefore be accompanied not only by adequate exposure to sunlight, but also by the oral intake of vitamin D supplements [10,11,52]. Vitamin D intake can be supplemented by including bread spreads, which are fortified with vitamin D by law in Poland, as well as voluntarily fortified products such as breakfast cereals, yoghurts, milk and milk drinks, homogenized cheese, milk desserts, instant cocoa drinks, juices and vegetable drinks in the diet [53,54]. Consuming vitamin D-rich food groups (fish and shellfish, eggs, fortified dairy products and cereals) was associated with a better vitamin status [48,51].

According to recent epidemiological studies, there is a severe vitamin D deficiency in Polish children and adolescents [12,18]. Despite this, the level of public knowledge about the role of vitamin D, its status, dietary sources (which is particularly evident among young people aged 18–25 years) and remedial measures are inadequate [55]. Interventions should therefore include education on how to optimize diets to increase the supply of vitamin D. More attention should be paid to the sufficient intake of vitamin D in adolescents who do not use vitamin D supplements or fortified milk products. The optimisation of the diet has not only contributed to an increase in the supply of vitamin D [50] and an improved vitamin D status [48,51], but has also led to a 54% reduction in global greenhouse gas emissions [50]. This is also important in terms of planetary climate change.

### 4.1. The Study’s Strengths and Limitations

This study was conducted on a large sample of male adolescents from all macroregions of Poland. This study provided significant data on vitamin D intake and its determinants in a population of Polish adolescents, which is of particular significance when formulating dietary strategies to augment the supply of this vitamin to recommended levels. The FFQ utilised in this study is distinguished by its high validity and a favourable evaluation of its validity and repeatability.

A limitation of this study is that the indirect method was used, i.e., data were collected using a questionnaire that was provided to the study participants online, with the adolescents completing it based on self-assessment and declaration. In addition, the retrospective nature of the study may have contributed to recall bias, which may have affected the accuracy and/or completeness of the reported intake data. However, this has significantly facilitated the dissemination of information to a considerable number of individuals. Moreover, due to the nationwide design of the study and the fact that the collected data, among others, concerned the consumption of fish, which is usually not consumed every day (rather occasionally in Polish conditions), the use of another method (e.g., 24 h diet reminder or 3-day diet record) would not provide reliable data.

A further limitation of the study is that the questionnaire did not include questions about the seasonality of food consumption, as the design of the tool did not allow for this. The questions concerned the consumption of specific food products in the year preceding the survey, regardless of the season. There was also no question about the reasons for eating or not eating fish. The inclusion of such questions could have, on the one hand, enriched the results and, on the other hand, helped to establish recommendations for increasing the consumption of fish as a rich source of vitamin D. However, the decisive factor here was the time that the male adolescents had to devote to completing the questionnaire. The addition of further questions would have resulted in a more protracted time to complete the questionnaire, which could have resulted in a lower response rate.

An additional limitation of this study is that participants were not asked about the reasons for not taking vitamin D supplements, and adolescents who declared taking these supplements did not have the opportunity to declare their dosage. The questionnaire also did not contain questions about exposure to sunlight and the consumption of food products voluntarily fortified with vitamin D, e.g., milk and dairy products, plant-based drinks, or fruit drinks.

Another limitation of this study is that it does not include results on the determination of vitamin D status in the study group, which would be impossible to do in such a large group of subjects. On the other hand, reducing the number of participants in the group in which the vitamin D status was determined would result in data of a fragmentary nature being obtained.

Finally, male adolescents participated in the study voluntarily; therefore, despite the representative nature of the sampling plan, due to the insufficient response rate in the study, the collected data do not come from a representative sample. Nevertheless, it was possible to collect data from all macroregions of Poland, and the analysis was carried out for over 3200 male adolescents.

### 4.2. Future Research Directions

In subsequent studies, the following sources of vitamin D should be given due consideration: exposure to sunlight, including outdoor activity; residing in regions of the world with sunlight levels that differ from those experienced in Poland (e.g., during vacation); dietary sources, including food products voluntarily fortified with vitamin D; and the supply of dietary supplements and medicines containing vitamin D, taking into account detailed data on the name of the preparation, dosage and regularity of administration. In order to improve an individual’s vitamin D status, it is imperative to determine the concentration of both the free and bound forms of vitamin 25(OH)D in the blood. On the one hand, it is imperative to establish a correlation between the data on vitamin D supply from all sources and the specific vitamin D status, and on the other, the health outcomes of the subjects. Such a correlation should encompass body composition, blood pressure and heart rate, indicators of lipid and carbohydrate metabolism, as well as calcium and phosphate metabolism.

## 5. Conclusions

In conclusion, dietary vitamin D intake in the studied cohort of Polish male adolescents was very low. This can be attributed to the limited supply of vitamin D from dietary sources, especially fish and fish products. The determinants of dietary vitamin D intake in the study participants were the amount and species of fish consumed, the macroregion of residence, the location of the school, nutritional status, and the use of vitamin D supplements. In contrast, vitamin D intake was not associated with age. This study highlights the urgent need to develop strategies (including nutrition education) to increase knowledge about the role of vitamin D and its dietary sources in order to increase fish consumption among adolescents and the supply of vitamin D in this group. Consequently, from a public health standpoint, it is recommended that interventions be implemented not only at the level of schools and adolescents’ families, but also by local and national authorities. These actions could include the routine monitoring of vitamin D status in adolescence, which would facilitate the earlier detection of deficiencies in high-risk groups, such as adolescents. This approach would facilitate timely intervention, thereby minimizing the adverse effects of these deficiencies and their progression to more serious health consequences. Further research is necessary to determine the total vitamin D intake in adolescents, considering all sources of this vitamin in relation to vitamin D status and health outcomes.

## Figures and Tables

**Table 1:** Study group characteristics.

Variable			*n*	%
Total			3257	100
Macroregion of Poland	Central		206	6.32
	Eastern		600	18.42
	Masovian Voivodeship		208	6.39
	Northern		1054	32.36
	North-Western		310	9.52
	Southern		549	16.86
	South-Western		330	10.13
Location of the school	Countryside		119	3.65
	Small city (<20,000)		776	23.83
	Medium city (20,000–100,000)		2142	65.77
	Big city (>100,000)		220	6.75
Age	14–17 years old		2445	75.07
	18–20 years old		812	24.93
Body Mass Index (BMI) classification	Underweight	All	163	5.00
		14–17 years old	118	4.82
		18–20 years old	45	5.54
	Normal weight	All	2249	69.05
		14–17 years old	1699	69.49
		18–20 years old	550	67.73
	Overweight	All	493	15.14
		14–17 years old	344	14.07
		18–20 years old	149	18.35
	Obesity	All	352	10.81
		14–17 years old	284	11.62
		18–20 years old	68	8.38
Vitamin D supplementation	No		1759	54.01
	Yes		1498	45.99

**Table 2:** Intake of vitamin D from different food groups in male adolescents.

Vitamin D Source			Dietary Vitamin D Intake in µg/day	
			Mean ± SD	Median (Min–Max)
Total ^1^			13.31 ± 18.86	4.36 (0.00–72.59)
Fish and fish product groups	Total	Total	10.18 ± 18.52	1.13 (0.00–67.53)
		All fish	9.60 ± 18.42	0.72 (0.00–65.69)
		All fish products	0.58 ± 1.32	0.00 (0.00–20.91)
	F/FP_GR1	Total	1.34 ± 2.61	0.02 (0.00–36.60)
		Salmon	0.60 ± 1.35	0.00 (0.00–20.28)
		Herring	0.28 ± 0.59	0.00 (0.00–7.50)
	F/FP_GR2		8.50 ± 18.26	0.08 (0.00–51.27)
	F/FP_GR3		0.04 ± 0.01	0.00 (0.00–1.40)
	F/FP_GR4		0.28 ± 0.74	0.00 (0.00–12.36)
	F/FP_GR5		0.02 ± 0.05	0.00 (0.00–0.93)
Other food groups	Dairy products		0.88 ± 2.03	0.39 (0.00–20.15)
	Eggs		0.94 ± 1.15	0.61 (0.00–8.71)
	Meat and meat products		0.99 ± 0.92	0.71(0.00–6.15)
	Cereals		0.19 ± 0.22	0.13 (0.00–2.28)
	Fats		0.13 ± 0.25	0.05 (0.00–3.07)

^1^ from all food sources; F/FP_GR1—salmon, rainbow trout, herring, eel; F/FP_GR2—halibut, mackerel, brook trout, sole, tuna; F/FP_GR3—cod, flounder, plaice, pollock, hake; F/FP_GR4—herring, sardine, and tuna products; F/FP_GR5—other fish products.

**Table 3:** Declared number of servings of different food groups consumed weekly or daily among male adolescents.

Food Groups			Servings	
			Mean ± SD	Median (Min–Max)
Fish and fish product groups (per week)	Total	Total	3.74 ± 4.92	2.10 (0.93–50.63)
		All fish	2.49 ± 4.48	0.93 (0.00–40.73)
		All fish products	1.25 ± 0.73	0.93 (0.93–12.60)
	F/FP_GR1	Total	0.92 ± 1.71	0.23 (0.00–21.23)
		Salmon	0.26 ± 0.55	0.00 (0.00–7.00)
		Herring	0.40 ± 0.88	0.00 (0.00–12.83)
	F/FP_GR2		0.57 ± 1.19	0.00 (0.00–14.00)
	F/FP_GR3		0.67 ± 1.64	0.00 (0.00–22.87)
	F/FP_GR4		0.28 ± 0.74	0.00 (0.00–12.36)
	F/FP_GR5		0.02 ± 0.05	0.00 (0.00–0.93)
Other food groups (per day)	Dairy products		3.68 ± 3.37	2.75 (0.00–28.71)
	Eggs		1.09 ± 1.33	0.71 (0.00–10.00)
	Meat and meat products		2.20 ± 1.88	1.71 (0.00–12.14)
	Cereals		4.23 ± 3.86	3.14 (0.00–28.57)
	Fats		1.64 ± 2.13	1.00 (0.00–25.57)

F/FP_GR1—salmon, rainbow trout, herring, eel; F/FP_GR2—halibut, mackerel, brook trout, sole, tuna; F/FP_GR3—Cod, flounder, plaice, pollock, hake; F/FP_GR4—herring, sardine, and tuna products; F/FP_GR5—other fish products.

**Table 4:** Daily vitamin D intake (µg) from all food groups in the subgroups of male adolescents.

Vitamin D Intake Determinants		Vitamin D Intake (µg/d)		*p* Value **
		Mean ± SD	Median (Min–Max) *	
Macroregions of Poland	C (*n* = 206)	5.34 ± 5.44	3.72 (0.00–33.01) ^A^	<0.0001
	E (*n* = 600)	16.45 ± 20.80	5.18 (0.00–66.20) ^B^	
	M (*n* = 206)	8.16 ± 13.00	3.94 (0.00–67.08) ^A^	
	N (*n* = 1054)	9.99 ± 16.00	3.59 (0.00–68.14) ^A^	
	N-W (*n* = 310)	35.27 ± 23.74	50.57 (0.00–71.60) ^C^	
	S (*n* = 549)	10.82 ± 16.38	4.24 (0.00–72.59) ^A^	
	S-W (*n* = 330)	10.01 ± 15.11	4.29 (0.00–68.57) ^A^	
Location of the school	C-S (*n* = 119)	30.00 ± 24.86	49.49 (0.00–68.14) ^A^	0.0003
	Sc (*n* = 776)	13.13 ± 18.91	4.15 (0.00–0.00) ^BC^	
	Mc (*n* = 2142)	13.17 ± 18.71	4.39 (0.00–72.59) ^B^	
	Bc (*n* = 220)	6.36 ± 8.26	3.97 (0.00–62.95) ^C^	
Age (years)	14–17 (*n* = 2445)	13.58 ± 19.18	4.29 (0.00–72.59)	0.271
	18–20 (*n* = 812)	12.51 ± 17.87	4.59 (0.00–71.60)	
Body Mass Index classification	Uw (*n* = 163)	9.29 ± 14.88	3.38 (0.00–57.41) ^A^	0.0007
	Nw (*n* = 2249)	13.97 ± 19.37	4.59 (0.00–71.60) ^B^	
	Ow (*n* = 493)	11.50 ± 17.21	4.10 (0.00–72.59) ^AB^	
	Ob. (*n* = 352)	13.53 ± 19.12	4.06 (0.00–62.35) ^AB^	
Vitamin D supplementation	No (*n* = 1759)	13.03 ± 18.86	4.06 (0.00–70.30) ^A^	0.0001
	Yes (*n* = 1498)	13.65 ± 18.87	4.71 (0.00–72.59) ^B^	

C—Central, E—Eastern, M—Masovian Voivodeship, N—Northern, N-W—North-Western, S—Southern, S-W—South-Western; C-S—countryside, Sc—small city, Mc—medium city, Bc—big city; Uw—underweight, Nw—normal weight, Ow—overweight, Ob.—obesity * the distribution was not parametric, as verified by the Shapiro–Wilk test; ** comparisons were made using Kruskal–Wallis test; values marked with different letters (A, B, C) in the columns are significantly different; *p* ≤ 0.05.

**Table 5:** Correlations between the declared weekly number of servings of fish and fish products and dVDi, and between Body Mass Index (BMI) and the total dVDi in a group of male adolescents.

Variable		Correlations	
		r	*p*
Weekly number of servings of fish and fish products per week vs. total dVDi	F/FP	0.68	<0.0001
	All fish	0.66	<0.0001
	F/FP_GR1	0.65	<0.0001
	F/FP_GR2	0.55	<0.0001
	F/FP_GR3	0.46	<0.0001
	F/FP_GR4 and F/FP_GR5	0.53	<0.0001
Vitamin D intake from fish and fish products (µg/day) vs. total dVDi	Intake from F/FP	0.81	<0.0001
	All fish	0.80	<0.0001
	F/FP_GR1	0.45	<0.0001
	F/FP_GR2	0.73	<0.0001
	F/FP_GR3	0.32	<0.0001
	F/FP_GR4 and F/FP_GR5	0.37	<0.0001
BMI (kg/m^2^) vs. total dVDi		0.001	0.9349

dVDi—dietary vitamin D intake; F/FP—total fish and fish products; F/FP_GR1—group of fish species containing 7–15 µg of vitamin D in 100 g of product, i.e., salmon, rainbow trout, herring, and eel; F/FP_GR2—group of fish species containing 1.05–4 µg of vitamin D in 100 g of product, i.e., halibut, mackerel, brook trout, sole, and tuna; F/FP_GR3—group of fish species containing 0.35–0.5 µg of vitamin D in 100 g of product, i.e., cod, flounder, plaice, pollock, and hake; F/FP_GR4—group of herring, sardine, and tuna products; F/FP_GR5—group of other fish products.

## Data Availability

Data available on request due to ethical reasons.

## Supplementary Materials

The following supporting information can be downloaded at: https://www.mdpi.com/article/10.3390/nu17122024/s1, Figure S1: VIDEO-FFQ-Vitamin D Estimation Only-Food Frequency Questionnaire; Figure S2: Percentage of male adolescents with dietary vitamin D intake below the AI level in different subgroups; Table S1: Daily vitamin D intake from various food groups in the subgroups of male adolescents from different macroregions of Poland; Table S2: Daily vitamin D intake from different food groups in subgroups of male adolescents by school location; Table S3: Daily vitamin D intake from different food groups in subgroups of male adolescents by age of participants; Table S4: Daily vitamin D intake from different food groups in subgroups of male adolescents by Body Mass Index (BMI) classification; Table S5: Daily vitamin D intake from different food groups in subgroups of male adolescents by vitamin D supplementation.

## Abbreviations

The following abbreviations are used in this manuscript:

dVDi	dietary vitamin D intake, intake of vitamin D from dietary sources
F/FP	total fish and fish products
F/FP_GR1	group of fish species containing 7–15 µg of vitamin D in 100 g of product, i.e., salmon, rainbow trout, herring, and eel
F/FP_GR2	group of fish species containing 1.05–4 µg of vitamin D in 100 g of product, i.e., halibut, mackerel, brook trout, sole, and tuna
F/FP_GR3	group of fish species containing 0.35–0.5 µg of vitamin D in 100 g of product, i.e., cod, flounder, plaice, pollock, and hake
F/FP_GR4	group of herring, sardine, and tuna products
F/FP_GR5	group of other fish products
VDD	vitamin D deficiency
