# Supplementary material for: Assessment of Determinants of Dietary Vitamin D Intake in a Polish National Sample of Male Adolescents
Source: Nutrients. 2025 Jun 17;17(12):2024. doi: 10.3390/nu17122024 (PMC12196299; doi:10.3390/nu17122024)
Supplement: Supplementary file 1 [file nutrients-17-02024-s001.zip › nutrients-3682786-supplementary.pdf]

*Supplementary Materials*

# Assessment of Determinants of Dietary Vitamin D Intake in a Polish National Sample of Male Adolescents

**Małgorzata Stachoń and Katarzyna Lachowicz**

Name/ code ……………………………………………………………………………………

*Please specify typical number of servings of consumed products and products added to consumed dishes (not only integers, but also decimal parts of servings).*

*Afterwards, please underline the most commonly chosen products from the groups of fresh and smoked fish and of fish products (1-2 products for each → indicated cell).*

| Group of products | Products | Serving size | Frequency | Number of servings |
|---|---|---|---|---|
| Fresh and smoked fish | → Salmon, rainbow trout, herring, eel | 50 g (deck of cards) | monthly | |
| | → Halibut, mackerel, brook trout, sole, tuna | 50 g (deck of cards) | monthly | |
| | → Cod, flounder, plaice, pollock, hake, bass, zander, pike | 50 g (deck of cards) | monthly | |
| Fish products | → Herrings, sardines and tuna products | 100 g (e.g. 2 rollmopses, small can of tuna, 2/3 of can of herrings) | monthly | |
| | → Other fish products | 100 g (e.g. 1/3 of can of fish stew) | monthly | |
| Dairy products | Milk and milk beverages (yoghurt, kefir, buttermilk, cream) | 250 g (1 glass) | weekly | |
| | Rennet cheese | 20 g (1 slice) | weekly | |
| | Blue and soft penicillium cheese | 150 g (1 package) | weekly | |
| | Feta cheese | 15 g (1 slice) | weekly | |
| | Cottage cheese | 50 g (1 thick slice, 2 tablespoons) | weekly | |
| | Processed cheese | 25 g (1 slice, 1 spoon, 1 triangle serving) | weekly | |
| | Homogenized cheese, dairy desert | 150 g (1 package) | weekly | |
| | Dairy ice cream | 40 g (1 scoop) | monthly | |
| Eggs | Egg | 50 g (1 medium egg) | weekly | |
| | Egg yolk | 20 g (1 yolk) | weekly | |
| Meat | | 100 g (palm of small hand) | weekly | |
| Meat products | | 15 g (thin slice of ham, 3 slices of sausage) | weekly | |
| Cereals | White wheat and confectionery bread | 35 g (1 slice, small roll) | weekly | |
| | Cooked egg pasta | 100 g of cooked (1 glass) | weekly | |
| Fats | Butter, butter products, pork fat | 5g (1 teaspoon) | daily | |
| | Margarine | 5g (1 teaspoon) | daily | |

**VIDEO-FFQ – Vitamin D Estimation Only – Food Frequency Questionnaire**
Głąbska D., Guzek D., Sidor P., Włodarek D. *Nutrients* 2016, 8(1), 36, doi:10.3390/nu8010036

**Figure S1.** VIDEO-FFQ-Vitamin D Estimation Only-Food Frequency Questionnaire.

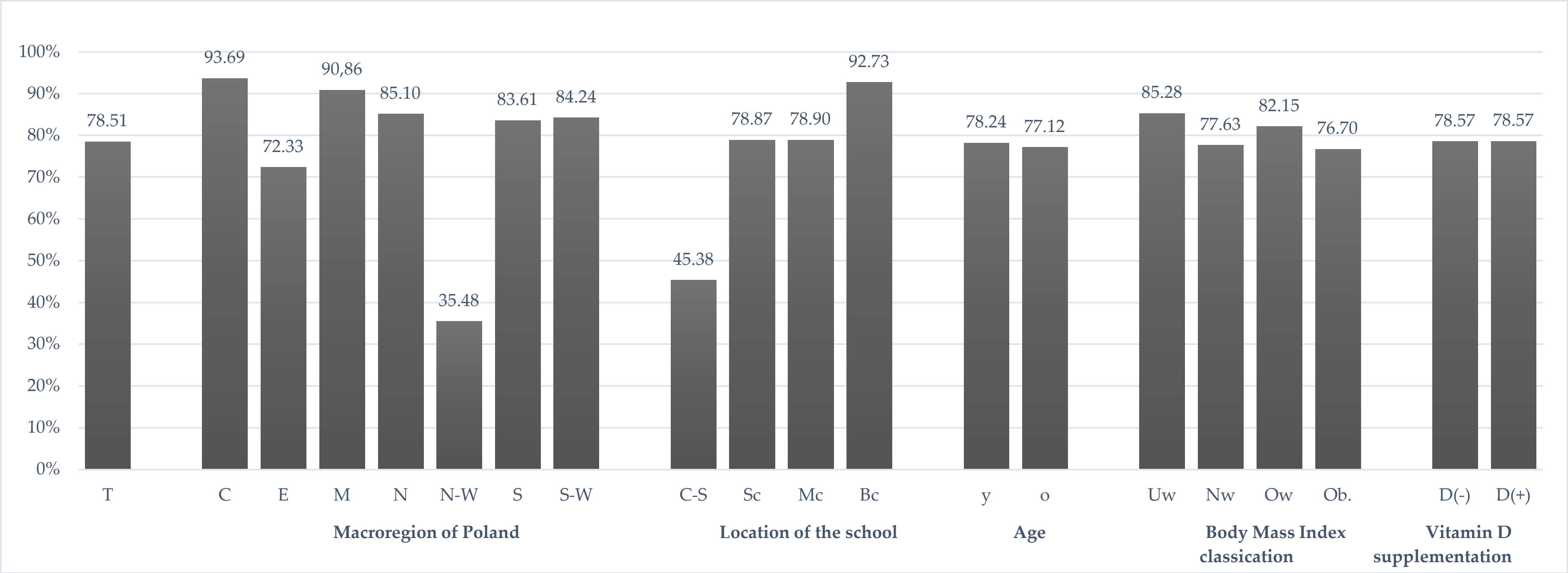


**Figure S2.** Percentage of male adolescents with dietary vitamin D intake below the AI level in different subgroups. (T – total, C – Central, E – Eastern, M – Masovian Voivodeship, N – Northern, N-W – North-Western, S – Southern, S-W – South-Western; C-S – countryside, Sc – small city, Mc – medium city, Bc – big city; y – 14 to 17 years old, o – 18 to 20 years old; Uw – underweight, Nw – normal weight, Ow – overweight, Ob – obesity; D(-) – not supplementing vitamin D, D(+) – supplementing vitamin D; AI – adequate intake).

**Table S1.** Daily vitamin D intake from various food groups in the subgroups of male adolescents from different macroregions of Poland.

| Vitamin D source | | Intake (µg) | Macroregions of Poland C (n=206) | E (n=600) | M (n=208) | N (n=1054) | N-W (n=310) | S (n=549) | S-W (n=330) | *p* Value ** |
|---|---|---|---|---|---|---|---|---|---|---|
| Fish and fish product groups | F/FP | Mean±SD | 1.91±3.59 | 13.51±20.68 | 5.30±12.64 | 7.06±15.66 | 31.50±23.67 | 7.24±15.88 | 7.20±14.75 | <0.0001 |
| | | Median* | 0.44 $^{A}$ | 1.71 $^{B}$ | 0.97 $^{AD}$ | 0.75 $^{AD}$ | 48.60 $^{C}$ | 0.98 $^{D}$ | 1.18 $^{BD}$ | |
| | | (Min-Max) | (0.00-26.68) | (0.00-59.36) | (0.00-63.48) | (0.00-63.95) | (0.00-64.80) | (0.00-67.53) | (0.00-61.89) | |
| | F/FP_GR1 | Mean±SD | 1.28±2.76 | 1.34±2.49 | 1.37±2.17 | 1.23±2.53 | 1.44±2.77 | 1.22±2.49 | 1.76±3.22 | 0.054 |
| | | Median* | 0.00 | 0.32 | 0.50 | 0.25 | 0.32 | 0.32 | 0.50 | |
| | | (Min-Max) | (0.00-22.16) | (0.00-17.92) | (0.00-10.65) | (0.00-29.29) | (0.00-20.15) | (0.00-36.62) | (0.00-21.74) | |
| | F/FP_GR2 | Mean±SD | 0.27±0.66 | 11.83±20.70 | 3.54±12.21 | 5.54±15.22 | 29.70±23.73 | 5.68±15.40 | 4.99±14.35 | <0.0001 |
| | | Median* | 0.00 $^{A}$ | 0.17 $^{B}$ | 0.06 $^{A}$ | 0.00 $^{A}$ | 48.60 $^{C}$ | 0.00 $^{A}$ | 0.08 $^{A}$ | |
| | | (Min-Max) | (0.00-6.50) | (0.00-51.27) | (0.00-50.29) | (0.00-51.00) | (0.00-50.88) | (0.00-51.00) | (0.00-49.08) | |
| | F/FP_GR3 | Mean±SD | 5.34±5.44 | 0.04±0.10 | 0.05±0.10 | 0.04±0.09 | 0.05±0.14 | 0.03±0.08 | 0.05±0.11 | 0.066 |
| | | Median* | 3.72 | 0.00 | 0.00 | 0.00 | 0.00 | 0.00 | 0.00 | |
| | | (Min-Max) | (0.00-33.01) | (0.00-0.80) | (0.00-0.64) | (0.00-0.72) | (0.00-1.40) | (0.00-0.90) | (0.00-0.09) | |
| | F/FP_GR4 | Mean±SD | 0.29±0.60 | 0.29±0.60 | 0.31±1.04 | 0.23±0.55 | 0.29±0.77 | 0.29±0.82 | 0.38±0.95 | 0.233 |
| | | Median* | 0.00 | 0.00 | 3.94 | 0.00 | 0.00 | 0.00 | 0.00 | |
| | | (Min-Max) | (0.00-4.12) | (0.00-4.12) | (0.00-12.36) | (0.00-6.18) | (0.00-8.67) | (0.00-12.36) | (0.00-12.36) | |
| | F/FP_GR5 | Mean±SD | 0.02±0.04 | 0.02±0.05 | 0.02±0.06 | 0.02±0.05 | 0.02±0.06 | 0.01±0.03 | 0.03±0.07 | 0.445 |
| | | Median* | 0.00 | 0.00 | 0.00 | 0.00 | 0.00 | 0.00 | 0.00 | |
| | | (Min-Max) | (0.00-0.28) | (0.00-0.31) | (0.00-0.62) | (0.00-0.93) | (0.00-0.46) | (0.00-0.31) | (0.00-0.68) | |
| Dairy products | | Mean±SD | 1.04±2.58 | 0.67±1.19 | 0.45±0.40 | 0.90±2.23 | 1.02±2.08 | 1.30±2.77 | 0.55±0.73 | <0.0001 |
| | | Median* | 0.44 $^{ABC}$ | 0.39 $^{ACD}$ | 0.35 $^{AC}$ | 0.35 $^{AC}$ | 0.42 $^{BD}$ | 0.47 $^{B}$ | 0.36 $^{C}$ | |
| | | (Min-Max) | (0.00-19.45) | (0.00-18.71) | (0.00-2.93) | (0.00-19.97) | (0.00-19.66) | (0.00-20.15) | (0.00-8.28) | |
| Eggs | | Mean±SD | 1.01±1.09 | 0.99±1.25 | 1.01±1.33 | 0.86±1.09 | 1.12±1.20 | 0.87±1.07 | 0.97±1.16 | 0.0007 |
| | | Median* | 0.61 $^{AB}$ | 0.61 $^{AB}$ | 0.61 $^{AB}$ | 0.50 $^{A}$ | 0.75 $^{B}$ | 0.50 $^{A}$ | 0.61 $^{AB}$ | |
| | | (Min-Max) | (0.00-5.27) | (0.00-8.71) | (0.00-8.71) | (0.00-8.23) | (0.00-8.71) | (0.00-7.50) | (0.00-7.50) | |

| | | | | | | | | | |
|---|---|---|---|---|---|---|---|---|---|
| Meat and meat products | Mean±SD | 1.00±0.92 | 0.95±0.92 | 1.08±0.92 | 0.88±0.82 | 1.20±1.08 | 1.08±1.03 | 0.97±0.83 | <0.0001 |
| | Median* | 0.73 AB | 0.68 AC | 0.85 BC | 0.66 A | 0.84 B | 0.75 BC | 0.72 AB | |
| | (Min-Max) | (0.00-4.97) | (0.00-5.42) | (0.00-0.85) | (0.00-6.15) | (0.00-5.75) | (0.00-5.81) | (0.00-4.68) | |
| Cereals | Mean±SD | 0.20±0.25 | 0.19±0.23 | 0.21±0.23 | 0.18±0.20 | 0.24±0.27 | 0.19±0.21 | 0.18±0.22 | 0.011 |
| | Median* | 0.13 AB | 0.13 AB | 0.14 AB | 0.12 A | 0.16 B | 0.14 AB | 0.12 AB | |
| | (Min-Max) | (0.00-1.79) | (0.00-2.28) | (0.00-1.41) | (0.00-1.77) | (0.00-1.86) | (0.00-1.77) | (0.00-1.57) | |
| Fats | Mean±SD | 0.16±0.29 | 0.13±0.25 | 0.12±0.27 | 0.11±0.20 | 0.20±0.36 | 0.13±0.25 | 0.13±0.23 | 0.0002 |
| | Median* | 0.05 AB | 0.05 AB | 0.04 A | 0.04 A | 0.08 B | 0.04 A | 0.05 AB | |
| | (Min-Max) | (0.00-2.30) | (0.00-2.76) | (0.00-3.07) | (0.00-2.43) | (0.00-2.97) | (0.00-2.21) | (0.00-1.64) | |

F/FP – total fish and fish products, F/FP_GR1 – group of fish species containing 7-15 μg of vitamin D in 100 g of product, i.e. salmon, rainbow trout, herring, and eel, F/FP_GR2 – group of fish species containing 1.05-4 μg of vitamin D in 100 g of product, i.e. halibut, mackerel, brook trout, sole, and tuna, F/FP_GR3 – group of fish species containing 0.35-0.5 μg of vitamin D in 100 g of product, i.e. cod, flounder, plaice, pollock, and hake, F/FP_GR4 – group of herring, sardine, and tuna products, F/FP_GR5 – group of other fish products; C – Central, E – Eastern, M – Masovian Voivodeship, N – Northern, N-W – North-Western, S – Southern, S-W – South-Western; * the distribution was not parametric, as verified by the Shapiro-Wilk test; ** comparisons were made using Kruskal-Wallis test; values marked with different letters (A, B, C, D) in the rows are significantly different; $p \leq 0.05$.

**Table S2.** Daily vitamin D intake from different food groups in subgroups of male adolescents by school location.

| Vitamin D source | | Intake (µg) | Location of the school C-S (n=119) | Sc (n=776) | Mc (n=2142) | Bc (n=220) | *p* Value ** |
|---|---|---|---|---|---|---|---|
| Fish and fish product groups | F/FP | Mean±SD | 26.79±24.71 | 10.01±18.49 | 10.05±18.41 | 3.13±7.00 | 0.0014 |
| | | Median* | 48.60 A | 1.06 B | 1.11 B | 0.77 B | |
| | | (Min-Max) | (0.00-63.95) | (0.00-63.48) | (0.00-67.53) | (0.00-53.42) | |
| | F/FP_GR1 | Mean±SD | 1.06±1.91 | 1.41±2.96 | 1.28±2.44 | 1.73±3.18 | 0.309 |
| | | Median* | 0.25 | 0.32 | 0.25 | 0.36 | |
| | | (Min-Max) | (0.00-11.19) | (0.00-36.62) | (0.00-22.27) | (0.00-22.16) | |
| | F/FP_GR2 | Mean±SD | 25.46±24.39 | 8.25±18.06 | 8.43±18.20 | 0.97±5.66 | <0.0001 |
| | | Median* | 48.60 A | 0.08 B | 0.08 B | 0.03 B | |
| | | (Min-Max) | (0.00-50.04) | (0.00-51.00) | (0.00-51.27) | (0.00-49.18) | |
| | F/FP_GR3 | Mean±SD | 0.03±0.07 | 0.05±0.11 | 0.04±0.10 | 0.06±0.12 | 0.103 |
| | | Median* | 0.00 | 0.00 | 0.00 | 0.00 | |
| | | (Min-Max) | (0.00-0.50) | (0.00-0.09) | (0.00-1.40) | (0.00-0.09) | |
| | F/FP_GR4 | Mean±SD | 0.23±0.47 | 0.28±0.79 | 0.27±0.73 | 0.34±0.71 | 0.261 |
| | | Median* | 0.00 | 0.00 | 0.00 | 0.00 | |
| | | (Min-Max) | (0.00-2.88) | (0.00-12.36) | (0.00-12.36) | (0.00-4.94) | |
| | F/FP_GR5 | Mean±SD | 0.01±0.03 | 0.02±0.04 | 0.02±0.05 | 0.03±0.07 | 0.002 |
| | | Median* | 0.00 A | 0.00 AB | 0.00 AB | 0.00 B | |
| | | (Min-Max) | (0.00-0.25) | (0.00-0.43) | (0.00-0.93) | (0.00-0.62) | |
| Dairy products | | Mean±SD | 0.94±2.40 | 0.85±2.01 | 0.89±2.00 | 0.88±2.15 | 0.623 |
| | | Median* | 0.37 | 0.37 | 0.39 | 0.38 | |
| | | (Min-Max) | (0.00-18.58) | (0.00-19.66) | (0.00-20.15) | (0.00-19.97) | |
| Eggs | | Mean±SD | 0.94±1.04 | 0.90±1.11 | 0.95±1.18 | 0.93±1.15 | 0.368 |
| | | Median* | 0.61 | 0.50 | 0.61 | 0.61 | |
| | | (Min-Max) | (0.00-6.21) | (0.00-8.71) | (0.00-8.71) | (0.00-7.50) | |
| Meat and meat products | | Mean±SD | 0.97±0.84 | 1.04±0.98 | 0.96±0.90 | 1.04±0.94 | 0.161 |
| | | Median* | 0.73 | 0.73 | 0.69 | 0.80 | |
| | | (Min-Max) | (0.00-4.67) | (0.00-5.81) | (0.00-6.15) | (0.00-5.74) | |
| Cereals | | Mean±SD | 0.19±0.18 | 0.19±0.22 | 0.19±0.23 | 0.20±0.23 | 0.333 |
| | | Median* | 0.15 | 0.13 | 0.13 | 0.14 | |
| | | (Min-Max) | (0.00-0.96) | (0.00-1.79) | (0.00-2.28) | (0.00-1.33) | |
| Fats | | Mean±SD | 0.13±0.21 | 0.12±0.22 | 0.13±0.26 | 0.16±0.34 | 0.545 |
| | | Median* | 0.05 | 0.05 | 0.05 | 0.05 | |
| | | (Min-Max) | (0.00-1.06) | (0.00-2.40) | (0.00-2.97) | (0.00-3.07) | |

F/FP – total fish and fish products, F/FP_GR1 – group of fish species containing 7-15 µg of vitamin D in 100 g of product, i.e. salmon, rainbow trout, herring, and eel, F/FP_GR2 – group of fish species containing 1.05-4 µg of vitamin D in 100 g of product, i.e. halibut, mackerel, brook trout, sole, and tuna, F/FP_GR3 – group of fish species containing 0.35-0.5 µg of vitamin D in 100 g of product, i.e. cod, flounder, plaice, pollock, and hake, F/FP_GR4 – group of herring, sardine, and tuna products, F/FP_GR5 – group of other fish products; C-S – countryside, Sc – small city, Mc – medium city, Bc – big city; * the distribution was not parametric, as verified by the Shapiro-Wilk test; ** comparisons were made using Kruskal-Wallis test; values marked with different letters (A, B) in the rows are significantly different; $p \leq 0.05$.

**Table S3.** Daily vitamin D intake from different food groups in subgroups of male adolescents by age of participants.

| Vitamin D source | | Intake (µg) | Age (years) 14-17 (n=2445) | Age (years) 18-20 (n=812) | *p* Value ** |
|---|---|---|---|---|---|
| Fish and fish product groups | F/FP | Mean±SD | 10.52±18.86 | 9.17±17.45 | 0.769 |
| | | Median* (Min-Max) | 1.08 (0.00-67.53) | 1.25 (0.00-64.80) | |
| | F/FP_GR1 | Mean±SD | 1.30±2.57 | 1.46±2.73 | 0.072 |
| | | Median* (Min-Max) | 0.25 (0.00-36.62) | 0.50 (0.00-22.27) | |
| | F/FP_GR2 | Mean±SD | 8.89±18.60 | 7.35±17.18 | 0.128 |
| | | Median* (Min-Max) | 0.12 (0.00-51.27) | 0.08 (0.00-50.40) | |
| | F/FP_GR3 | Mean±SD | 0.04±0.10 | 0.04±0.11 | 0.767 |
| | | Median* (Min-Max) | 0.00 (0.00-1.40) | 0.00 (0.00-0.90) | |
| | F/FP_GR4 | Mean±SD | 0.27±0.74 | 0.30±0.73 | 0.137 |
| | | Median* (Min-Max) | 0.00 (0.00-12.36) | 0.00 (0.00-12.36) | |
| | F/FP_GR5 | Mean±SD | 0.02±0.05 | 0.02±0.04 | 0.356 |
| | | Median* (Min-Max) | 0.00 (0.00-0.93) | 0.00 (0.00-0.31) | |
| Dairy products | | Mean±SD | 0.89±1.99 | 0.87±2.14 | 0.116 |
| | | Median* (Min-Max) | 0.38 (0.00-20.15) | 0.38 (0.00-19.47) | |
| Eggs | | Mean±SD | 0.90±1.14 | 1.05±1.20 | <0.0001 |
| | | Median* (Min-Max) | 0.61 [A] (0.00-8.71) | 0.62 [B] (0.00-7.50) | |
| Meat and meat products | | Mean±SD | 0.96±0.89 | 1.07±1.01 | 0.053 |
| | | Median* (Min-Max) | 0.69 (0.00-5.81) | 0.77 (0.00-6.15) | |
| Cereals | | Mean±SD | 0.19±0.21 | 0.21±0.26 | 0.086 |
| | | Median* (Min-Max) | 0.13 (0.00-2.28) | 0.14 (0.00-1.86) | |
| Fats | | Mean±SD | 0.13±0.25 | 0.14±0.26 | 0.154 |
| | | Median* (Min-Max) | 0.05 (0.00-3.07) | 0.05 (0.00-2.97) | |

F/FP – total fish and fish products, F/FP_GR1 – group of fish species containing 7-15 µg of vitamin D in 100 g of product, i.e. salmon, rainbow trout, herring, and eel, F/FP_GR2 – group of fish species containing 1.05-4 µg of vitamin D in 100 g of product, i.e. halibut, mackerel, brook trout, sole, and tuna, F/FP_GR3 – group of fish species containing 0.35-0.5 µg of vitamin D in 100 g of product, i.e. cod, flounder, plaice, pollock, and hake, F/FP_GR4 – group of herring, sardine, and tuna products, F/FP_GR5 – group of other fish products; * The distribution was not parametric, as verified by the Shapiro-Wilk test; ** comparisons were made using the U Mann-Whitney test; $p \leq 0.05$.

**Table S4.** Daily vitamin D intake from different food groups in subgroups of male adolescents by Body Mass Index (BMI) classification.

| Vitamin D source | | Intake (μg) | Body Mass Index classification Uw (n=163) | Nw (n=2249) | Ow (n=493) | Ob (n=352) | *p* Value ** |
|---|---|---|---|---|---|---|---|
| Fish and fish product groups | F/FP | Mean±SD | 6.70±14.33 | 10.73±19.06 | 8.57±16.90 | 10.54±18.70 | 0.311 |
| | | Median* (Min-Max) | 1.05 (0.00-53.18) | 1.16 (0.00-64.80) | 1.00 (0.00-67.53) | 1.30 (0.00-57.84) | |
| | F/FP_GR1 | Mean±SD | 1.52±2.96 | 1.32±2.61 | 1.38±2.63 | 1.27±2.044 | 0.810 |
| | | Median* (Min-Max) | 0.32 (0.00-22.16) | 0.32 (0.00-36.62) | 0.32 (0.00-20.15) | 0.25 (0.00-16.67) | |
| | F/FP_GR2 | Mean±SD | 4.76±14.06 | 9.09±18.78 | 6.83±16.65 | 8.82±18.50 | 0.215 |
| | | Median* (Min-Max) | 0.03 (0.00-49.06) | 0.12 (0.00-51.27) | 0.08 (0.00-50.04) | 0.10 (0.00-50.25) | |
| | F/FP_GR3 | Mean±SD | 0.06±0.18 | 0.04±0.10 | 0.04±0.10 | 0.04±0.10 | 0.874 |
| | | Median* (Min-Max) | 0.00 (0.00-0.09) | 0.00 (0.00-1.40) | 0.00 (0.00-0.09) | 0.08 (0.00-0.00) | |
| | F/FP_GR4 | Mean±SD | 0.34±0.72 | 0.26±0.57 | 0.29±0.97 | 0.38±1.18 | 0.177 |
| | | Median* (Min-Max) | 0.00 (0.00-4.12) | 0.00 (0.00-8.65) | 0.00 (0.00-12.36) | 0.00 (0.00-12.36) | |
| | F/FP_GR5 | Mean±SD | 0.02±0.08 | 0.02±0.05 | 0.02±0.04 | 0.03±0.06 | 0.688 |
| | | Median* (Min-Max) | 0.00 (0.00-0.93) | 0.00 (0.00-0.68) | 0.00 (0.00-0.28) | 0.00 (0.00-0.46) | |
| Dairy products | | Mean±SD | 0.68±1.85 | 0.93±2.13 | 0.74±1.62 | 0.83±1.93 | <0.0001 |
| | | Median* (Min-Max) | 0.30 A (0.00-19.66) | 0.41 B (0.00-20.15) | 0.36 A (0.00-18.58) | 0.33 A (0.00-18.87) | |
| Eggs | | Mean±SD | 0.71±0.79 | 0.97±1.18 | 0.92±1.12 | 0.89±1.16 | 0.022 |
| | | Median* (Min-Max) | 0.49 A (0.00-4.29) | 0.61 B (0.00-8.71) | 0.61 AB (0.00-7.50) | 0.50 AB (0.00-8.71) | |
| Meat and meat products | | Mean±SD | 0.86±0.91 | 1.00±0.92 | 0.99±0.93 | 0.97±0.93 | 0.052 |
| | | Median* (Min-Max) | 0.60 (0.00-5.74) | 0.72 (0.00-6.15) | 0.72 (0.00-5.61) | 0.67 (0.00-5.36) | |
| Cereals | | Mean±SD | 0.18±0.19 | 0.20±0.23 | 0.18±0.21 | 0.19±0.23 | 0.178 |
| | | Median* (Min-Max) | 0.12 (0.00-1.06) | 0.13 (0.00-2.28) | 0.13 (0.00-1.57) | 0.12 (0.00-1.41) | |
| Fats | | Mean±SD | 0.14±0.26 | 0.13±0.26 | 0.12±0.24 | 0.11±0.19 | 0.0002 |
| | | Median* (Min-Max) | 0.05 A (0.00-2.21) | 0.05 A (0.00-3.07) | 0.04 B (0.00-2.76) | 0.04 B (0.00-1.36) | |

F/FP – total fish and fish products, F/FP_GR1 – group of fish species containing 7-15 μg of vitamin D in 100 g of product, i.e. salmon, rainbow trout, herring, and eel, F/FP_GR2 – group of fish species containing 1.05-4 μg of vitamin D in 100 g of product, i.e. halibut, mackerel, brook trout, sole, and tuna, F/FP_GR3 – group of fish species containing 0.35-0.5 μg of vitamin D in 100 g of product, i.e. cod, flounder, plaice, pollock, and hake, F/FP_GR4 – group of herring, sardine, and tuna products, F/FP_GR5 – group of other fish products; Uw – underweight, Nw – normal weight, Ow – overweight, Ob – obesity; * the distribution was not parametric, as verified by the Shapiro-Wilk test; ** comparisons were made using Kruskal-Wallis test; values marked with different letters (A, B) in the rows are significantly different; $p \leq 0.05$.

**Table S5.** Daily vitamin D intake from different food groups in subgroups of male adolescents by vitamin D supplementation.

| Vitamin D source | | Intake (μg) | Vitamin D supplementation No (n=1759) | Vitamin D supplementation Yes (n=1498) | *p* Value ** |
|---|---|---|---|---|---|
| Fish and fish product groups | F/FP | Mean±SD | 10.03±18.47 | 10.36±18.59 | 0.0002 |
| | | Median* | 0.94 [A] | 1.37 [B] | |
| | | (Min-Max) | (0.00-64.00) | (0.00-67.53) | |
| | F/FP_GR1 | Mean±SD | 1.30±2.73 | 1.38±2.46 | <0.0002 |
| | | Median* | 0.25 [A] | 0.50 [B] | |
| | | (Min-Max) | (0.00-36.62) | (0.00-22.95) | |
| | F/FP_GR2 | Mean±SD | 8.40±18.20 | 8.62±18.34 | <0.0001 |
| | | Median* | 0.00 [A] | 0.12 [B] | |
| | | (Min-Max) | (0.00-51.27) | (0.00-51.27) | |
| | F/FP_GR3 | Mean±SD | 0.04±0.11 | 0.04±0.09 | 0.010 |
| | | Median* | 0.00 [A] | 0.00 [B] | |
| | | (Min-Max) | (0.00-1.40) | (0.00-0.07) | |
| | F/FP_GR4 | Mean±SD | 0.27±0.74 | 0.29±0.74 | 0.120 |
| | | Median* | 0.00 | 0.00 | |
| | | (Min-Max) | (0.00-12.36) | (0.00-12.36) | |
| | F/FP_GR5 | Mean±SD | 0.02±0.05 | 0.02±0.05 | 0.618 |
| | | Median* | 0.00 | 0.00 | |
| | | (Min-Max) | (0.00-0.68) | (0.00-0.93) | |
| Dairy products | | Mean±SD | 0.86±2.18 | 0.90±1.84 | <0.0001 |
| | | Median* | 0.35 [A] | 0.44 [B] | |
| | | (Min-Max) | (0.00-20.15) | (0.00-19.45) | |
| Eggs | | Mean±SD | 0.88±1.09 | 1.01±1.22 | 0.0002 |
| | | Median* | 0.50 | 0.61 | |
| | | (Min-Max) | (0.00-8.71) | (0.00-8.71) | |
| Meat and meat products | | Mean±SD | 0.94±0.90 | 1.04±0.94 | 0.0003 |
| | | Median* | 0.68 [A] | 0.74 [B] | |
| | | (Min-Max) | (0.00-6.15) | (0.00-5.81) | |
| Cereals | | Mean±SD | 0.18±0.22 | 0.21±0.23 | 0.0001 |
| | | Median* | 0.12 | 0.14 | |
| | | (Min-Max) | (0.00-2.28) | (0.00-1.93) | |
| Fats | | Mean±SD | 0.14±0.27 | 0.12±0.23 | 0.677 |
| | | Median* | 0.05 | 0.05 | |
| | | (Min-Max) | (0.00-3.07) | (0.00-2.49) | |

F/FP – total fish and fish products, F/FP_GR1 – group of fish species containing 7-15 μg of vitamin D in 100 g of product, i.e. salmon, rainbow trout, herring, and eel, F/FP_GR2 – group of fish species containing 1.05-4 μg of vitamin D in 100 g of product, i.e. halibut, mackerel, brook trout, sole, and tuna, F/FP_GR3 – group of fish species containing 0.35-0.5 μg of vitamin D in 100 g of product, i.e. cod, flounder, plaice, pollock, and hake, F/FP_GR4 – group of herring, sardine, and tuna products, F/FP_GR5 – group of other fish products; * The distribution was not parametric, as verified by the Shapiro-Wilk test; ** comparisons were made using the U Mann-Whitney test; values marked with different letters (A, B) in the rows are significantly different; $p \leq 0.05$.
